# Supplementary material for: Evaluating the applicability of replication success metrics in animal-to-human translation: A simulation study
Source: eLife. 2026 Aug 7;15:RP109853. doi: 10.7554/eLife.109853 (PMC13451019; doi:10.7554/eLife.109853)
Supplement: Peer review history

# Editor report: eLife Assessment

**DOI:** 10.7554/eLife.109853.3.sa0

**Authors:** George N Okoli

**Affiliations:** University of Hong Kong Hong Kong

**Keywords:** Convincing, Important

This is a detailed and well-designed simulation study of the utility of replication metrics in animal-to-human study translations in bridging the gap between laboratory discoveries and health practice, a critical consideration in turning laboratory scientific research findings into tangible, real-world applications, to directly help human health. The study approaches are **convincing**, and the findings are **important**, as they offer insights into clinical research translations to advance health decision-making.

# Referee report: Reviewer #1 (Public review)

**DOI:** 10.7554/eLife.109853.3.sa1

[Editors' note: This revised version of your article has been assessed by the Reviewing Editor without further input from the original reviewers. The comments raised by the original reviewers in the earlier round of review have been addressed. The study findings are quite insightful and important, and the evidence is strong, convincing, and a substantial addition to the evidence base.]

A well-designed and preregistered simulation study investigating whether replication-success metrics can be applied to assess animal-to-human translation. The study is comprehensive, uses realistic parameter settings, and provides valuable insights into how different metrics behave under varied conditions.

Strengths:

(1) Methodologically rigorous and transparently preregistered.

(2) Comprehensive simulation design covering a wide range of plausible scenarios.

(3) Clear description of metrics and decision rules.

(4) Valuable contribution to understanding the limitations of applying replication metrics to translation questions.

# Referee report: Reviewer #2 (Public review)

**DOI:** 10.7554/eLife.109853.3.sa2

Summary:

The authors attempt to address the issue of high rates of translation failure from animal studies to humans in the literature, where promising results in animal studies fail when conducting human clinical trials. Using parameters from a previous meta-analysis on prenatal amino acid supplementation and the effects it has on maternal blood pressure, the authors assessed the performance of the metrics used and whether they can quantify translation success. Performing a simulation study, the authors compared nine translation success metrics and found that no one method was uniformly optimal. The authors list several limitations of the study, such as comparability of effect sizes between animal and human studies, different goals of animal studies versus human studies, and the focus of the study on one aspect (statistics of translation) is part of a broader, more complex decision-making process before proceeding to human trials. The authors recommend using multiple metrics in combination while taking into consideration their strengths and weaknesses to assess the translation of animal studies to human outcomes. The paper achieves the aim of providing a model with several metrics to evaluate translation success from animal studies to humans.

Strengths:

(1) Utilizing 9 different translation success metrics in combination provides strong flexibility in evaluating whether results in animal studies can translate to humans. This would allow researchers to evaluate translation success using multiple different metrics according to the context of the study.

(2) The authors accommodated for the limited sample size in animal studies, which are typically underpowered, and also caution that special attention should be given to heterogeneity when interpreting translation results.

(3) Overall, this approach has the potential to be applied to other biomedical studies, provided the limitations for each of the metrics are considered. It would provide a useful tool in assessing translation from animals to humans, in addition to other factors such as safety, pharmacokinetics, etc.

Weaknesses:

While the study has several strengths, there are some limitations.

(1) Preclinical animal study sizes tend to be much smaller than human studies, which results in underpowered results. The authors adjusted for this by pooling animal study data. However, high heterogeneity in the animal studies can affect translation results.

(2) The study focuses only on evaluating the statistical component of translation, which is only one aspect of the decision-making process to move on to human trials. The study does not take into account safety and toxicological profiles, pharmacokinetics, or genetics, which are important considerations that influence the overall effect in humans.

# Referee report: Reviewer #3 (Public review)

**DOI:** 10.7554/eLife.109853.3.sa3

Summary:

This paper focused on how to navigate the complex decision-making process of whether to go into human trials. This is a critical topic considering the well-documented challenges in replicating and translating findings. While these are two distinct topics (i.e., replication and translation), they are related, and the authors simulated many conditions to assess the utility of replication assessment metrics.

Strengths:

A major strength of the study is the detailed approach to identifying relevant conditions and metrics, and to providing rich results that outline the strengths and weaknesses of each metric. Any simulation study is challenged by trying to identify the most relevant variables of interest, and this study provided sound justification for its chosen variables of interest. While this study does not make a strong recommendation (which I see as a strength), it does provide a comprehensive overview of the various metrics and conditions that were investigated.

Conclusion:

This paper provides a much-needed investigation and discussion of how decisions are made when assessing whether to go into human trials. This is an important topic that productively challenges the status quo, considering documented challenges in replication and translation in biomedical research.

# Author comment: Author response

**DOI:** 10.7554/eLife.109853.3.sa4

**Authors:** Carolyne Jie Huang, Samuel Pawel, Kimberley E Wever, Benjamin Victor Ineichen, Rachel Heyard

**Affiliations:** University Hospital of Zurich Zurich Switzerland; University of Zurich Zurich Switzerland; Radboud University Medical Center Nijmegen Netherlands; University of Bern Bern Switzerland; University of Zurich Zürich Switzerland

The following is the authors’ response to the original reviews.

**Public Reviews:**

**Reviewer #1 (Public Review):**

Summary:

A well-designed and preregistered simulation study investigating whether replication-success metrics can be applied to assess animal-to-human translation. The study is comprehensive, uses realistic parameter settings, and provides valuable insights into how different metrics behave under varied conditions.

Strengths:

(1) Methodologically rigorous and transparently preregistered.

(2) Comprehensive simulation design covering a wide range of plausible scenarios.

(3) Clear description of metrics and decision rules.

(4) Valuable contribution to understanding the limitations of applying replication metrics to translation questions.

Weaknesses:

(1) The conceptual distinction between replication and translation could be more clearly emphasized.

(2) Interpretation of results is dense and can be challenging to follow without a clear and summarized.

(3) Some simulation parameters (effect sizes, heterogeneity, and number of animal studies) require more substantial justification.

(4) Practical recommendations could be more explicit to guide applied researchers.

We thank Reviewer 1 for the general positive assessment of our study and for the constructive feedback. We have addressed all of the four identified weaknesses in the revised manuscript. Specifically,

(1) Conceptual distinction between replication and translation. We have reinforced this distinction at multiple points in the manuscript: in Section 2.7 (just before introducing the translation success metrics), in the Discussion, and in a new working definition of translation success added to the Introduction. We further explicitly acknowledge that statistical translation success, as defined here, is narrower than biological translation.

(2) The dense result section. We have added a summary Table (Table 3) at the end of the Results section that compares all metrics on key properties (strengths and weaknesses, overall type 1 error control, sensitivity to heterogeneity, dependence on animal sample size and number of studies, and behaviour under effect mismatch). We also direct readers to this table early in Section 3.2, so that readers less interested in the technical details can obtain the key take-home messages without reading the full section.

(3) Further justification of simulation parameters. We have substantially extended the rationale for our parameter choices in Section 2.4 and the Limitations section. We explain that our parameters are grounded in an empirical meta-analytic dataset, contextualise the large effect size and heterogeneity value against published benchmarks from preclinical research, and clarify that our main goal was to explore directional trends rather than absolute performance under specific values. We have also added an invitation for others to explore alternative parameter spaces using our openly available code.

(4) Practical recommendations. We have extended the Recommendations section (pages 21–22) with more explicit scenario-specific guidance, supported by the new summary table.

**Reviewer #2 (Public review):**

Summary:

The authors attempt to address the issue of high rates of translation failure from animal studies to humans in the literature, where promising results in animal studies fail when conducting human clinical trials. Using parameters from a previous meta-analysis on prenatal amino acid supplementation and the effects it has on maternal blood pressure, the authors assessed the performance of the metrics used and whether they can quantify translation success. Performing a simulation study, the authors compared nine translation success metrics and found that no one method was uniformly optimal. The authors list several limitations of the study, such as comparability of effect sizes between animal and human studies, different goals of animal studies versus human studies, and the focus of the study on one aspect (statistics of translation) is part of a broader, more complex decision-making process before proceeding to human trials. The authors recommend using multiple metrics in combination while taking into consideration their strengths and weaknesses to assess the translation of animal studies to human outcomes. The paper achieves the aim of providing a model with several metrics to evaluate translation success from animal studies to humans.

Strengths:

(1) Utilizing 9 different translation success metrics in combination provides strong flexibility in evaluating whether results in animal studies can translate to humans. This would allow researchers to evaluate translation success using multiple different metrics according to the context of the study.

(2) The authors accommodate for the limited sample size in animal studies, which are typically underpowered, and also caution that special attention should be given to heterogeneity when interpreting translation results.

(3) Overall, this approach has the potential to be applied to other biomedical studies, provided the limitations for each of the metrics are considered. It would provide a useful tool in assessing translation from animals to humans, in addition to other factors such as safety, pharmacokinetics, etc.

Weaknesses:

While the study has several strengths, there are some limitations.

(1) Preclinical animal study sizes tend to be much smaller than human studies, which results in underpowered results. The authors adjusted for this by pooling animal study data. However, high heterogeneity in the animal studies can affect translation results.

(2) The study focuses only on evaluating the statistical component of translation, which is only one aspect of the decision-making process to move on to human trials. The study does not take into account safety and toxicological profiles, pharmacokinetics, or genetics, which are important considerations that influence the overall effect in humans.

We thank Reviewer 2 for the thoughtful summary and for recognising the strengths of our study. We believe that both weaknesses were addressed in the revised version of our manuscript. Specifically,

(1) Heterogeneity in animal studies. We agree that high heterogeneity in animal studies is an important limitation, and we address it directly in our simulation design by including a wide range of heterogeneity values (including very high levels, as observed in animal studies). Our results show clearly how heterogeneity affects the performance of each metric, and we highlight this in both the new summary Table (Table 3) and the Recommendations section which was extended. We also caution applied researchers to pay special attention to heterogeneity when interpreting translation results.

(2) Focus on the statistical component of translation. We fully agree that statistical translation success is only one aspect of a broader decision-making process. We have elaborated on this in the revised manuscript, both in a new working definition of translation success in the Introduction (which explicitly distinguishes statistical from biological translation) and in a new paragraph in the Discussion section where we situate our metrics within translational decision-making frameworks such as PATH. They make it clear that progression to human trials depends on a suite of evidence of which statistical translation is only one part.

**Reviewer #3 (Public review):**

Summary:

This paper focused on how to navigate the complex decision-making process of whether to go into human trials. This is a critical topic considering the well-documented challenges in replicating and translating findings. While these are two distinct topics (i.e., replication and translation), they are related, and the authors simulated many conditions to assess the utility of replication assessment metrics.

Strengths:

A major strength of the study is the detailed approach to identifying relevant conditions and metrics, and to providing rich results that outline the strengths and weaknesses of each metric. Any simulation study is challenged by trying to identify the most relevant variables of interest, and this study provided sound justification for its chosen variables of interest. While this study does not make a strong recommendation (which I see as a strength), it does provide a comprehensive overview of the various metrics and conditions that were investigated.

Weaknesses:

The weaknesses of the study are the limited focus on specific metrics, the assumptions, particularly in the limited number of human study variables, and the less-than-ideal approachable summary of findings for a non-technical audience.

Conclusion:

This paper provides a much-needed investigation and discussion of how decisions are made when assessing whether to go into human trials. This is an important topic that productively challenges the status quo, considering documented challenges in replication and translation in biomedical research.

We thank Reviewer 3 for the positive assessment and for the constructive suggestions.

We have addressed the identified weaknesses as follows:

(1) The assumptions around human study variables. We acknowledge these as inherent constraints of the simulation design. We have added a note in the Limitations section about the fixed human sample size (N = 107 per group), clarifying that while this value is grounded in a power analysis as per regulatory standards, it represents one particular scenario and may not generalise to all contexts. Further, we have contextualised and motivated the other simulation parameters better. We also invite readers to explore alternative conditions using our openly available code.

(2) Approachability of the summary of findings for a non-technical audience. We have added a summary Table (Table 3) at the end of the Results section, comparing the metrics on key properties including overall type 1 error control, sensitivity to heterogeneity, dependence on animal sample size and number of studies, and behaviour under effect mismatch. We direct readers to this table early in Section 3.2 so that those less interested in the technical details can obtain the main take-home messages without reading the full section.

**Recommendations for the authors:**

**Reviewer #1 (Recommendations for the authors):**

Major points:

(1) Conceptual framing: clearer distinction between replication vs translation

The Introduction correctly points out the conceptual difference between replication and translation (animal to human), but this distinction needs to be reinforced repeatedly, especially when interpreting metric performance. For instance, several metrics (e.g., meta-analysis, replication BF) inherently assume exchangeability of findings, which is rarely justified in translation because species differ biologically.

The manuscript should explicitly state why treating animal findings as "original studies" and human findings as "replications" can be misleading. Add a subsection in the Discussion: Why replication metrics behave differently in translation settings. This will help provide a more straightforward interpretation of the results beyond the numerical findings.

Thank you for your feedback. While the purpose of our study is to assess the applicability of the replication success metrics in the translation context, we agree that the reader should be reminded that these two concepts differ and metrics’ assumptions might not always hold. We have reiterated the difference between replication and translation in Section 2.7, just before we introduce the translation success metrics (see end of page 7). We also reiterate it in the Discussion (see end of page 20). Here, we emphasise that while some of the metrics assume that both studies investigate the same effect, this is unlikely to be the case in translation, leading to some of the metric’s assumptions being violated which impacts the performance of the metrics.

(2) Stronger justification of the simulation parameters is needed

The simulation factors are comprehensively presented (Table 1), but certain choices appear arbitrary or oversimplified.

- Effect sizes: The three levels (0, −4.44, −24.37) are derived from the motivating dataset, but the paper should explain that these represent extremely large effects in many biomedical contexts.

- Heterogeneity values: τ^2^ = 291.1 is enormous; adding context about real-world heterogeneity distributions would help.

- Number of animal studies (k): Using only 2-5 studies may not reflect reality; many preclinical fields have >30 studies before clinical translation.

These choices should be more explicitly defended in Section 4 (Limitations), beyond the brief mention already there. Provide a sensitivity analysis, or explain why extrapolation beyond this parameter space is reasonable.

We agree that the choice of the parameter values might sometimes appear arbitrary. However, instead of arbitrarily choosing parameter values, we base our choice on data from a meta-analysis. This particular meta-analysis might not be representative of all of pre-clinical and clinical research, but because Terstappen included both animal and human studies investigating the same research question it was particularly well suited. They further used an outcome (maternal blood pressure) that is comparable between rats and humans, which is quite rare. We have specified this further in Section 2.4 (Motivating dataset, page 5). In the Limitations section, we acknowledge any possibly unrealistic simulation conditions again, and emphasize that our main goal was to explore trends in the metrics’ behavior as the conditions changed rather than their absolute performance under specific values. Further, the effect sizes (0, −4.44, −24.37 mmHg) span a meaningful range on the unstandardized mean difference scale for blood pressure measurements: from no effect to a modest but clinically relevant reduction to a large effect typical of animal studies. The large heterogeneity value corresponds to a relative heterogeneity of I^2 of 95.33% in the animal meta-analysis. While this appears high, it is frequently observed in preclinical research: Hooijmans et al (2022) showed that 55% of animal study meta-analyses using mean differences as effect size measure have I^2>75%. We also added a footnote reiterating the fact that such high effect sizes (on the raw mean difference scale) are indeed common in animal studies (on page 6). Regarding k, we acknowledge that pooling only 2 to 5 animal studies may not reflect common practice. However, the directional trends in type 1 error and power are clearly visible in our Figures. Larger k decreases the type 1 error of the animal studies, while the power is increased unless there is high heterogeneity between animal studies and there is only a small effect. Extending the range further is unlikely to change the conclusions. Moreover, in practice, the decision to advance to human trials considers evidence well beyond the statistical considerations we simulate. All of the above is now emphasized more explicitly in both the methods, where we have substantially extended the reasoning for choosing the simulation conditions, and the limitations section. Finally, we added an invitation to others to use our open material (i.e., code) and explore the behaviour of the metrics under other conditions (see top of page 21).

(3) Decision criteria (strict/lenient/no criterion) need a clearer rationale

The three continuation rules are a strength of the study, but:

- The lenient criterion (any negative estimate is considered "beneficial") is unrealistic and should be reframed.

- The strict criterion (p < 0.025) heavily inflates effect sizes (in Figure 1b) and may distort interpretation.

It would be helpful to provide a table showing, for each criterion, its real-world analogue (e.g., regulatory requirement, exploratory progression, mechanistic plausibility).

We have followed your suggestion and added a Table (Table 2) with the description of the criterion and a description of its real-world analogue. No criterion represents an important reference scenario used to evaluate metric behaviour independent of progression decisions. The strict criterion is the closest to regulatory-style evidence. It is also highly selective and therefore might induce biases (e.g., inflated effect sizes). We link lenient to an exploratory decision-making where efficacy evidence is considered in addition to other factors (e.g., safety), but not intended to represent a certain regulatory standard.

(4) Interpretation of simulation results needs more focus

The Results section is extremely detailed, making it challenging to identify the central take-home messages. The authors should consider adding a concise summary table comparing metrics on key properties:

- T1E control robustness.

- Sensitivity to heterogeneity.

- Dependence on animal sample size.

- Dependence on k.

- Bias under asymmetric effects.

Moving some nested-loop plot descriptions to the Supplement. Right now, descriptions are technically correct but cognitively heavy.

We agree with your comment and have attempted to implement it in our summary Table 3, at the end of the results section. We also point readers early on to the Table, so that they can skip the more technical and detailed description if they want (see first paragraph section 3.2, page 12). After some trial and error, we agreed that the chosen columns are the most useful for an applied researcher to get a quick overview. Our table now summarises for each metric its main strengths and weaknesses, its behaviour with increasing heterogeneity, its sensitivity to more animal data (i.e., larger k and larger animal sample size), and its behaviour under effect mismatch (i.e., when the true effect in the animal and human study are dissimilar).

(5) The discussion should provide explicit recommendations.

The authors provide high-level recommendations, but the recommendations lack specific guidance. When heterogeneity is low, controlled sceptical p-value works well. When effect sizes differ: weighted Edgington is stable. The authors should avoid using replication BF when the animal effect ≠ human effect. Meta-analysis should not be used when human heterogeneity is high, because of inflated T1E.

We agree that explicit recommendations would be helpful to the applied researcher. As mentioned in the reply to the previous comment, we have added a summary table which lists the strengths and weaknesses of each metric. We also extended the paragraph in the Recommendations section (on page 21 and 22) to give some examples of scenarios in which certain metrics would be recommended over others.

(6) Recommendations for applied researchers

The study is missing an explicit definition of "translation success". The manuscript implicitly defines translation success as: "Both animal and human results show a beneficial treatment effect according to metric X". But this is different from biological translation, which concerns underlying mechanisms. The authors briefly mention this conceptual challenge, but this should be elaborated, as it is central to interpretation.

Thank you for this comment. We agree that “translation success” was not explicitly defined. We have now added a working definition in the Introduction, clarifying that, in this paper, translation success is defined statistically, and depends on the metric. We now explicitly acknowledge that this is a narrower definition than biological translation. We also elaborate on this distinction in the Discussion where we note that the appropriate metric and interpretation of translation success depends on the translation goal and that statistical translation is distinct from biological translation.

Minor points:

(1) The abstract could include a direct sentence on the main conclusion. For example, no metric was uniformly optimal; controlled sceptical p-value and weighted Edgington performed most consistently.

Our abstract already included main conclusions. We added the word “However” to emphasize the sentence “no metric was uniformly optimal” a bit more.

(2) The figures are informative, but nested loop plots are very dense. Consider providing a guided example in the figure caption explaining how to read them (as partially done in Figure 1a, but repeat for all).

We agree that the Figures can be very overwhelming at first. We did not want to add specific helping elements as we did in Figure 1 to not make the figures even busier. The goal was to introduce the reader gently to the nested loop plots via Figure 1 before having them look at the remaining figures. We hope that with the added summary Table and the more detailed recommendations, applied researchers less interested in the statistical details will still find the information most relevant for them easily.

(3) Methods: Section 2.4 could clearly state that effect sizes are in units of mmHg (blood pressure) from the dataset.

Thank you for pointing this out. This has been added.

(4) Results: This section is long; consider adding a brief summary paragraph at the end of 3.2.

We added a summary table, allowing interested readers to skip the long section entirely.

(5) Limitations: Add a note about publication bias in animal studies (you mention it in the Introduction, but not in Limitations). Add a statement about effect direction consistency (i.e., animal effect negative but human positive), which is not explored in the simulation grid.

Thank you for pointing out this inconsistency. A note about publication bias in animal studies was added to the Limitations section (that this was not investigated). A note about opposite animal and human effects was added to Section 2.5 (Simulation conditions) under “Animal and human effect sizes”.

**Reviewer #2 (Recommendations for the authors):**

Animal studies are typically highly controlled, using animal models that are either outbred to provide higher genetic variability or inbred with very little genetic variability and with a specific phenotype. Additionally, many rodent models are incomplete models of the overall human phenotype and are typically used to investigate only one aspect of the condition/disease. Some of the rat animal models that the Terstappen et al. (2020) systematic review used as the simulation parameters for the study included outbred (Sprague-Dawley, Wistar) and inbred Spontaneous Hypertensive Rats (SHR), which have different mechanisms in which hypertensive onset can occur, especially if inducing preeclampsia in outbred animals. Is it feasible to reduce heterogeneity in the animal results if only outbred or only SHR are considered instead? I realize this may reduce the sample size even further.

You raise an important point differentiating biological (rather than statistical) translation. We have added a sentence about differences between rat models and humans to the new paragraph in the Limitations section (bottom page 20 and top page 21) on the distinction between biological and statistical translation. As for reducing heterogeneity in the animal results by focusing on one type of rats, we agree focusing on one type of rats might reduce heterogeneity. We however consider this reduction to be very small (because the results of the study with SHR are actually comparable to the results with Wistar and SD rats). Therefore, rerunning the simulation would not yield results that differ in any meaningful way from those already reported and the substantial computational effort required to do so is not warranted.

**Reviewer #3 (Recommendations for the authors):**

Overall, I found this a very detailed study. However, my recommendation is to provide a more approachable overview of the results to reach a wider audience. Currently, the article is much more technical and statistically focused. I think two additions could help.

(1) A summary table of each of the metrics and their strengths and weaknesses under the various conditions (e.g., animal and human study characteristics). Currently, this is done via text, but I think a high-level summary via a table could be a compelling way to make the simulations more approachable for a non-technical audience.

As requested also by reviewer 1, we have added a summary table.

(2) Contextualize the findings within the decision-making process a little more. The authors have a well-written limitations section that acknowledges this; however, I think the discussion (and maybe the introduction) could be enriched by putting the simulation findings into context. For example, this paper suggests a framework that includes replication as part of the decision-making process for human trials (https://www.cell.com/med/fulltext/S2666-6340(24)00296-4).

We agree that situating our metrics within existing translational decision-making frameworks adds important context. We have added a paragraph in the Discussion (before the Limitations section on page 21) clarifying that the metrics evaluated here should not be viewed as standalone decision rules for progression from animal studies to human trials. Several frameworks have recently emerged precisely to guide such decisions in a more structured, multidimensional way. We refer to PATH and also to the GALENOS approach [DOI: 10.1186/s12874-026-02891-4]. Within such frameworks, translation success metrics of the kind evaluated here may provide a quantitative assessment of the consistency between animal and human efficacy findings, thereby informing one component of a broader translational evidence assessment. We have also briefly mentioned at the end of the Introduction (page 4) that frameworks for structuring the use of preclinical evidence in translational decisions are being developed, further motivating the need for quantitative tools such as those evaluated here.

Below are some additional minor comments for the authors to consider:

(1) In the abstract (4th line), there is an extra 'l' in failure.

Thank you for the detailed review. We have fixed this.

(2) I think since the study is completed, the objectives in the introduction should be past tense, not future.

We have fixed this.

(3) The limitations section should include the fixed human sample size. N=107 per group is grounded in the literature, but this varies widely based on the effect size of interest. Again, not material to the point of translation under simulated conditions (of which this would have increased the simulations well above the 648 already included), but given the impact this has on insights, this limits this investigation to a degree and should be acknowledged.

Thank you for your comment. We have added a note about the human sample size to the paragraph about the simulation conditions in the Limitations section. The human sample size was computed via power analysis as per regulations, but we realize this could change depending on the effect size.

(4) I appreciate how shrinkage was calculated. Though it is worth noting that the Reproducibility Project: Cancer Biology found much higher rates, which are similar to reports from biotech and pharma (e.g., 11% and 20-25% for Amgen and Bayer).

We already mentioned the high rates of shrinkage in the Replication Project Cancer Biology (see page 10). We have now also emphasised that one could adapt these levels further depending on the situation.
